# Filtering de novo indels in parent-offspring trios

**DOI:** 10.1186/s12859-020-03900-z

**Published:** 2020-12-16

**Authors:** Yongzhuang Liu, Jian Liu, Yadong Wang

**Affiliations:** School of Computer Science and Technology, Harbin Institute of Technology, 92 West Dazhi Street, Harbin, 150001 China

**Keywords:** Gradient boosting, Machine learning, De novo indel

## Abstract

**Background:**

Identification of de novo indels from whole genome or exome sequencing data of parent-offspring trios is a challenging task in human disease studies and clinical practices. Existing computational approaches usually yield high false positive rate.

**Results:**

In this study, we developed a gradient boosting approach for filtering de novo indels obtained by any computational approaches. Through application on the real genome sequencing data, our approach showed it could significantly reduce the false positive rate of de novo indels without a significant compromise on sensitivity.

**Conclusions:**

The software DNMFilter_Indel was written in a combination of Java and R and freely available from the website at https://github.com/yongzhuang/DNMFilter_Indel.

## Background

Spontaneous de novo germline indels were demonstrated to cause many human complex and rare diseases [1, 2]. With the rapid advancement of genome sequencing technology, the parent-offspring trio-based whole genome and exome sequencing is widely adopted for detecting de novo indels in clinical diagnosis and genetic studies [3, 4]. In general, de novo indels are usually identified by standard methods and joint calling methods. The standard method refers to that commmly used indel detection methods [5–7] are firstly employed to detect indels for all individuals in a trio independently and putative de novo indels are then identified by comparing the genotypes of parents and the offspring. The joint calling methods refers to direct detection of de novo indels from the trio, and representative methods include DeNovoGear [8], PhaseByTransmission [9] and TrioDeNovo [10]. Because de novo indels are exceedingly rare (2.94 indels per individual) [11] and the false discovery rate of current de novo indel detection methods is significantly higher than the indel mutation rate, a very small amount of true de novo indels are usually mixed with a large number of false ones. Therefore, effective de novo indel filtering methods are urgently needed.

Here, we present DNMFilter_Indel, a de novo indel filtering method that extends from our previous work DNMFilter [12]. Firstly, we integrate local de novo assembly to refine the alignment. Secondly, we add the classification model with two new sequence features strongly related to de novo indels. Additionally, to expand the positive set, we simulate synthetic de novo indels which can overcome the problem of the limited number of cross validated de novo indels. Finally, we evaluate DNMFilter_Indel’s performance using the real sequencing data of a whole genome trio from 1000 Genomes Project.

## Implementation

**Fig. 1:**
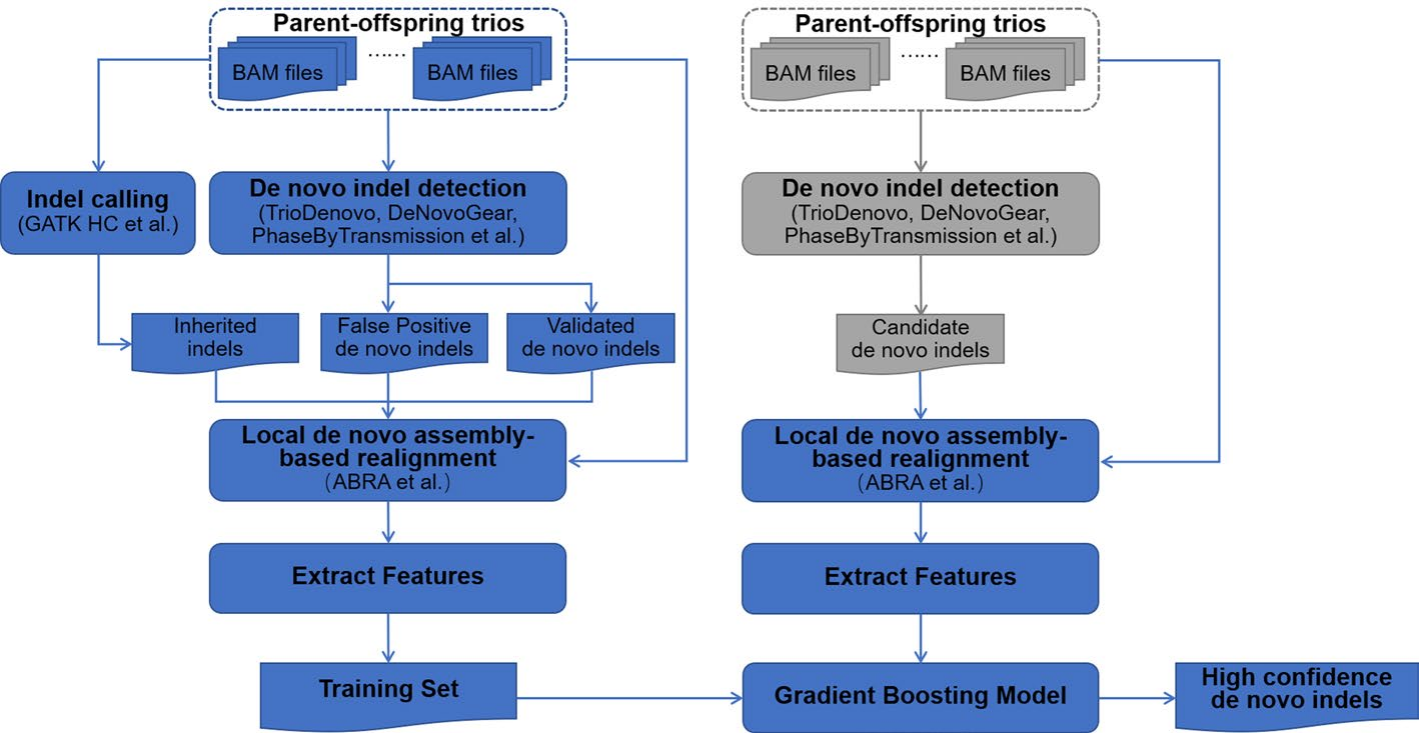
The pipeline of DNMFilter_Indel

The DNMFilter_Indel pipeline comprises two main modules: (a) Training; (b) Prediction, which is shown in Fig. 1.

In the Training module, firstly, DNMFilter_Indel takes the trios’ alignment files as input and employs commomly used de novo indel detection methods, such as DeNovoGear [8], PhaseByTransmission [9] and TrioDeNovo [10], to detect de novo indels; secondly, DNMFilter_Indel detects inherited indels using state-of-the-art indel detection methods (e.g. GATK HaplotypeCaller [5]); thirdly, DNMFilter_Indel uses the synthetic and cross validated de novo indels as positive examples and random sampling false de novo indels and inherited indels as negative examples; finally, DNMFilter_Indel performs local de novo assembly to refine the alingment for any positive or negative example, and then extracts sequence features from the refined alignment data to construct a training set.

In the Prediction module, DMFilter_Indel uses the same gradient boosting classification model as DNMFilter [12] to train the model and makes predictions for all putative de novo indels obtained via any computational methods. DNMFilter_Indel finally produces a score of 0 to 1 for each de novo indel, which represents the possibility of classification as real de novo indel.

### Sequence feature selection

Indel detection is more prone to alignment errors, so some commonly used indel detection methods do local de novo assembly to refine the alignment around candidate indels, and then detect indels from the realignment pileups. In order to correct alignment errors, DNMFilter_Indel uses the same strategy to perform local de novo assembly using ABRA2 [13] and extracts all sequence features for any de novo indel when training and predicting.

A large number of indels are from homopolymer and short tandem repeat (STR) regions of the human genome, but meanwhile indel detection is more prone to errors in homopolymer and STR regions. Hence, in addition to the sequence features used in DNMFilter, DNMFilter_Indel includes two additional sequence features to the classification model. One sequence feature is homopolymer, which refers to the repetitive sequence element with a unit of 1bp (the minimum repeat tract is set to 4); the other is short tandem repeat, which refers to the repetitive sequence elements with a unit of 2bp to 6bp (the minimum repeat tract is set to 3).

### Training set construction

Considering that de novo indel mutation rate is extremely low, it is hard to gather sufficient true de novo indels with cross validation as the positive examples. Here, we simulate synthetic de novo indels to complement the number of true de novo indels. The simulating process is as below. If one parent’s genotype is reference and the other parent’s genotype is a heterozygous indel, and at the same time the offspring’s genotype is reference, then the alingment information of the parent carrying the heterozygous indel and the offspring are exchanged. The exchanged indel sites can be regarded as synthetic de novo indel sites. The false de novo indels are produced according to the following process: (a) several commonly used de novo indel detection methods are run to get putative de novo indels; (b) the cross validated de novo indels are excluded; (c) the false de novo indels are randomly sampled from the set got by the previous step. Besides, inherited indels are also included as the negative examples.

## Results

The widely used CEU trio from 1000 Genomes Project is adopted to demostrate the performance of DNMFilter_Indel. The whole genome alignment files were got from ftp://ftp.1000genomes.ebi.ac.uk/vol1/ftp/technical/working/20120117_ceu_trio_b37_decoy/. All reads were mapped to human reference genome (GRCh37). There are 56 de novo indels in the CEU trio that were previously cross validated [8].

The training set was constructed with chromosome 1 to chromosome 6 of the trio, including 2000 positive examples (30 validated and 1970 synthetic de novo indels) and 4000 negative examples (2000 random sampling false de novo indels and 2000 inherited indels). Three state-of-the-art de novo indel detection methods, including DeNovoGear, PhaseByTransmission and TrioDeNovo, were adopted to detect de novo indels in the remaining chromosome 7 to chromosome 22, and DNMFilter_Indel was then employed to filter out false de novo indels obtained by these detection methods separately. DeNovoGear, PhaseByTransmission and TrioDeNovo were all run with default settings, and DNMFilter_Indel’s score cutoff was set to 0.4. DNMFilter_Indel was applied both on the raw alignment data and the refined alignment data based on local de novo assembly.

**Fig. 2:**
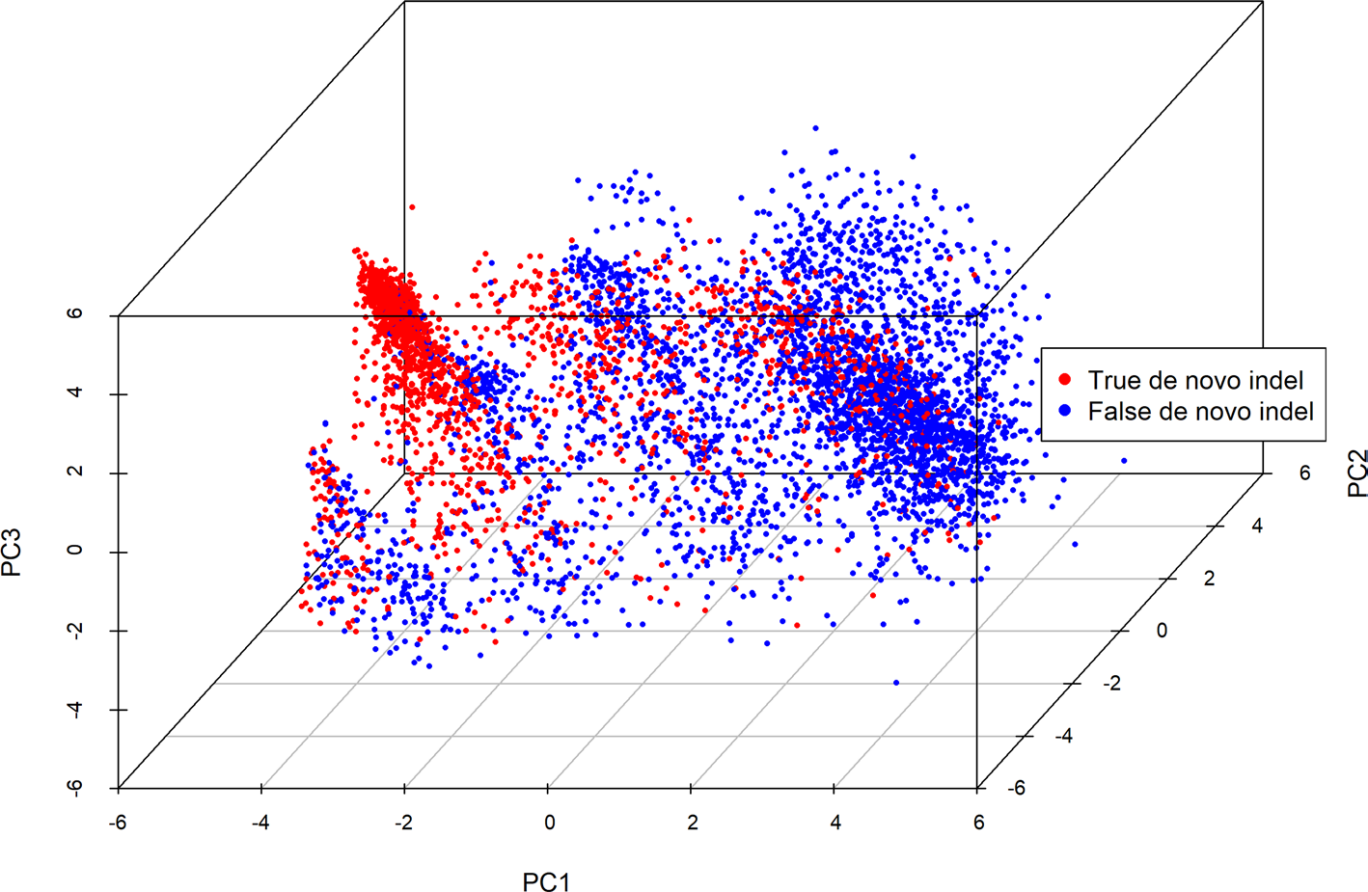
PCA analysis for the training set

**Fig. 3:**
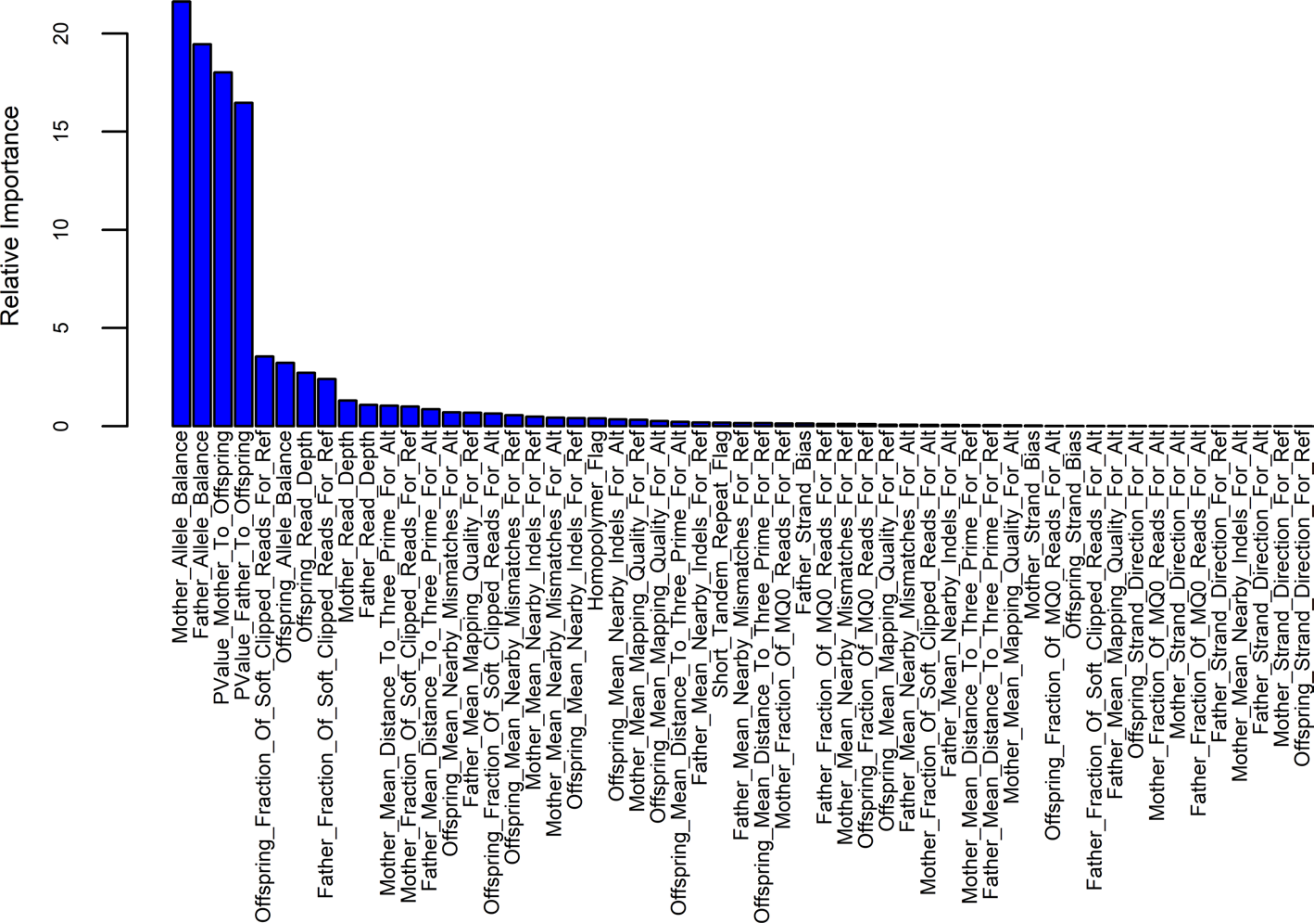
Feature importance ranking of all sequence features used

Foe the training set, the principal component analysis (PCA) was performed to project all sequence features of de novo indels to first three components (Fig. 2), and the result suggested that the sequence features used in this study were able to distinguish between true and false de novo indels. The feature importance ranking meansures were performed using the method provided in the R package “gbm” to determine the contribution of sequence features (Fig. 3). The result suggested that homopolymer and STR that we introduced ranked 21st and 27th respectively, indicating that two new sequence features introduced were useful for the classification.

The overall performance of DNMFilter_Indel coupled with de novo detection methods was illustrated in Table 1. The results showed that DNMFilter_Indel substantially filtered out false de novo indels with almost no loss in sensitivity. For any de novo indel detction method coupled with DNMFilter_Indel, only one true de novo indel was filtered out by mistake on the raw alignment data; no de novo indel was filtered out by mistake on the refined alignment data based on local de novo assembly. Too many remaining de novo indels in the final results may be due to that a lot of true de novo indels were not cross validated in the previous study. In conclusion, local de novo assembly-based refined alingment was effective for improving filtering performace; the positive set consisting of both the validated and synthetic de novo indels was effective for filtering de novo indels.

**Table 1:** Performance of DNMFilter_Indel used with three state-of-the-art de novo indel detection methods

Approaches	Without filtering		DNMFilter_Indel without local assembly		DNMFilter_Indel with local assembly	
	Sensitivity (%)	Number	Sensitivity (%)	Number	Sensitivity (%)	Number
PhaseByTransmission	92	583	88	182	92	197
DenovoGear	84	217	80	114	84	122
TrioDeNovo	92	3267	88	328	92	325

## Conclusions

We proposed a novel method DNMFilter_Indel extended from our previous work DNMFilter, which can effectively filter de novo indels from the trio-based sequencing data. By applying on the real sequencing data, DNMFilter_Indel is shown it could substantially filtered out false de novo indels with hardly sacrificing sensitivity. Together with the tool, the training set constructed with the CEU trio used in this study is released. The researchers can directly use this training set or construct a new training set with the module provided in DNMFilter_Indel, and then use DNMFilter_Indel to get true de novo indels mixed with a massive number of false ones.

## Availability and requirements

- Project name: DNMFilter_Indel.
- Project home page: https://github.com/yongzhuang/DNMFilter_Indel
- Operating system: Linux dependent.
- Programming language: Java and R.
- License: MIT.
- Any restrictions to use by non-academics: licence needed.

## Data Availability

The alignment files of the CEU trio are available at ftp://ftp.1000genomes.ebi.ac.uk/vol1/ftp/technical/working/20120117_ceu_trio_b37_decoy/. The cross validated de novo indels are available at Supplementary Table 9 of the DenovoGear paper (https://www.nature.com/articles/nmeth.2611).

## Abbreviations

STR: Short tandem repeat
PCA: Principal component analysis

## References

[1] Dong S, Walker MF, Carriero NJ, DiCola M, Willsey AJ, Ye AY, Waqar Z, Gonzalez LE, Overton JD, Frahm S, Keaney JF, Teran NA, Dea J, Mandell JD, Bal VH, Sullivan CA, DiLullo NM, Khalil RO, Gockley J, Yuksel Z, Sertel SM, Ercan-Sencicek AG, Gupta AR, Mane SM, Sheldon M, Brooks AI, Roeder K, Devlin B, State MW, Wei L, Sanders SJ (2014). De novo insertions and deletions of predominantly paternal origin are associated with autism spectrum disorder. Cell Rep.

[2] Fromer M, Pocklington A, Kavanagh D, Williams HJ, Dwyer S, Gormley P, Georgieva L, Rees E, Palta P, Ruderfer D, Carrera N, Humphreys I, Johnson JS, Roussos P, Barker DD, Banks E, Milanova V, Grant SG, Hannon E, Rose SA, Chambert K, Mahajan M, Scolnick EM, Moran JL, Kirov G, Palotie A, McCarroll SA, Holmans PA, Sklar P, Owen MJ, Purcell SM, O’Donovan MC (2014). De novo mutations in schizophrenia implicate synaptic networks. Nature.

[3] Need AC, Shashi V, Hitomi Y, Schoch K, Shianna KV, McDonald MT, Meisler MH, Goldstein DB (2012). Clinical application of exome sequencing in undiagnosed genetic conditions. J Med Genet.

[4] Turner TN, Hormozdiari F, Duyzend MH, McClymont SA, Hook PW, Iossifov I, Raja A, Baker C, Hoekzema K, Stessman HA, Zody MC, Nelson BJ, Huddleston J, Sandstrom R, Smith JD, Hanna D, Swanson JM, Faustman EM, Bamshad MJ, Stamatoyannopoulos J, Nickerson DA, McCallion AS, Darnell R, Eichler EE (2016). Genome sequencing of autism-affected families reveals disruption of putative noncoding regulatory dna. Am J Hum Genet.

[5] Poplin R, Ruano-Rubio V, DePristo MA, Fennell TJ, Carneiro MO, der Auwera GAV, Kling DE, Gauthier LD, Levy-Moonshine A, Roazen D, Shakir K, Thibault J, Chandran S, Whelan C, Lek M, Gabriel S, Daly MJ, Neale B, MacArthur DG, Banks E. Scaling accurate genetic variant discovery to tens of thousands of samples. bioRxiv, 201178 2017.

[6] Garrison E, Marth G. Haplotype-based variant detection from short-read sequencing. arXiv preprint arXiv:1207.3907. 2012.

[7] Rimmer A, Phan H, Mathieson I, Iqbal Z, Srf T, Aom W, McVean G, Lunter G (2014). Integrating mapping-, assembly- and haplotype-based approaches for calling variants in clinical sequencing applications. Nat Genet.

[8] Ramu A, Noordam MJ, Schwartz RS, Wuster A, Hurles ME, Cartwright RA, Conrad DF (2013). Denovogear: de novo indel and point mutation discovery and phasing. Nat Methods.

[9] Francioli LC, Cretu-Stancu M, Garimella KV, Fromer M, Kloosterman WP, Samocha KE, Neale BM, Daly MJ, Banks E, DePristo MA, de Bakker PI (2017). A framework for the detection of de novo mutations in family-based sequencing data. Eur J Hum Genet.

[10] Wei Q, Zhan X, Zhong X, Liu Y, Han Y, Chen W, Li B (2015). A bayesian framework for de novo mutation calling in parents-offspring trios. Bioinformatics.

[11] Kloosterman WP, Francioli LC, Marschall T, Hehir-Kwa JY, Abdellaoui A, Lameijer E-W, Moed MH, Koval V, Renkens I, van Roosmalen MJ, Arp P, Karssen LC, Coe BP, Handsaker RE, Suchiman ED, Cuppen E, Thung DT, McVey M, Wendl MC, van Duijn CM, Swertz MA, van Ommen GB, Slagboom PE, Boomsma DI, Schönhuth A, Eichler EE, Guryev V (2015). Characteristics of de novo structural changes in the human genome. Genome Res.

[12] Liu Y, Li B, Tan R, Zhu X, Wang Y (2014). A gradient-boosting approach for filtering de novo mutations in parent—offspring trios. Bioinformatics.

[13] Mose LE, Perou CM, Parker JS (2019). Improved indel detection in dna and rna via realignment with abra2. Bioinformatics.

